# An online randomised controlled trial of prognosticating imminent death in advanced cancer patients: Clinicians give greater weight to advice from a prognostic algorithm than from another clinician with a different profession

**DOI:** 10.1002/cam4.5485

**Published:** 2022-11-29

**Authors:** Andrea Bruun, Nicola White, Linda Oostendorp, Victoria Vickerstaff, Adam J. L. Harris, Christopher Tomlinson, Steven Bloch, Patrick Stone

**Affiliations:** ^1^ Division of Psychiatry, Marie Curie Palliative Care Research Department University College London London United Kingdom; ^2^ The Research Department of Primary Care and Population Health Institute of Epidemiology and Health Care, University College London London United Kingdom; ^3^ Division of Psychology and Language Sciences, Department of Experimental Psychology University College London London United Kingdom; ^4^ Department of Metabolism, Digestion and Reproduction, Faculty of Medicine Imperial College London London United Kingdom; ^5^ Division of Psychology and Language Sciences, Department of Language and Cognition University College London London United Kingdom

**Keywords:** behavioural science, judge‐advisor system, metastasis, neoplasms, prognosis, prognostic algorithm, randomised controlled trial

## Abstract

**Background:**

A second opinion or a prognostic algorithm may increase prognostic accuracy. This study assessed the level to which clinicians integrate advice perceived to be coming from another clinician or a prognostic algorithm into their prognostic estimates, and how participant characteristics and nature of advice received affect this.

**Methods:**

An online double‐blind randomised controlled trial was conducted. Palliative doctors, nurses and other types of healthcare professionals were randomised into study arms differing by perceived source of advice (algorithm or another clinician). In fact, the advice was the same in both arms (emanating from the PiPS‐B14 prognostic model). Each participant reviewed five patient summaries. For each summary, participants: (1) provided an initial probability estimate of two‐week survival (0% ‘certain death’—100% ‘certain survival’); (2) received advice (another estimate); (3) provided a final estimate. Weight of Advice (WOA) was calculated for each summary (0 ‘100% advice discounting’ – 1 ‘0% discounting’) and multilevel linear regression analyses were conducted.

Clinical trial registration number: NCT04568629.

**Results:**

A total of 283 clinicians were included in the analysis. Clinicians integrated advice from the algorithm more than advice from another clinician (WOA difference = −0.12 [95% CI ‐0.18, −0.07], *p* < 0.001). There was no interaction between study arm and participant profession, years of palliative care or overall experience. Advice of intermediate strength (75%) was given a lower WOA (0.31) than advice received at either the 50% (WOA 0.40) or 90% level (WOA 0.43). The overall interaction between strength of advice and study arm on WOA was significant (*p* < 0.001).

**Conclusion:**

Clinicians adjusted their prognostic estimates more when advice was perceived to come from a prognostic algorithm than from another clinician. Research is needed to understand how clinicians make prognostic decisions and how algorithms are used in clinical practice.

## 1 INTRODUCTION

It is important for clinicians to develop and maintain skills in predicting survival when a disease has become incurable and disease‐directed treatment options are limited or non‐existent.^1^ Accurate prognoses about how long patients have left to live can facilitate patient‐centred care and shared decision‐making.^2^ It is important and relevant to recognise when patients are close to death because discharge planning, discussions about resuscitation status, goals of care, hospice transfers and use of integrated care plans are all dependent on a patient's prognosis.^3^ However, clinicians' survival estimates are often inaccurate and over‐optimistic.^4^, ^5^, ^6^, ^7^

The European Association for Palliative Care (EAPC) recommends that a second opinion might be useful for improving prognostic accuracy.^8^ Another recommendation is that, when possible, a discussion with a multi‐professional team should be conducted as this may help refine the prognostic estimate.^1^ Evidence shows some improvement in accuracy when prognoses are formulated by multidisciplinary teams.^9^, ^10^ Other than maximising prognostic accuracy, doctors may also seek advice for social reasons (including self‐affirmation and sharing of responsibility^11^) and because of concerns about the consequences of making inaccurate predictions.^12^, ^13^ Another recommendation by the EAPC is that clinical prediction of survival should be used in conjunction with other prognostic factors.^8^ Several prognostic tools and algorithms have been developed and validated for use in advanced cancer,^14^, ^15^ but none have been found to be consistently superior to an agreed multi‐professional estimate of survival.^16^

Since the recommendations by the EAPC mention that clinicians should seek information, or ‘advice’, from colleagues or prognostic algorithms when making survival predictions, it is relevant to investigate how and to which degree prognostic advice from either of these sources is taken into account in a final survival prediction. Evidence suggests that human barriers to algorithm adoption are substantial in clinical practice,^17^ and there is conflicting evidence about whether people generally prefer advice from humans or algorithms.^18^, ^19^, ^20^ Concerns have been expressed about prognostic models leading to overconfidence or excessive prognostic certainty.^21^ Additionally, the outputs of prognostic algorithms may be difficult to interpret or lack face validity.^15^, ^17^ Some prognostic algorithms may also be unsuitable for use in patients with advanced disease where there can be issues with for instance obtaining required blood samples, which may not be appropriate if a patient is close to death.^22^

Expertise has previously been shown to have an impact on advice‐taking, with experts tending to discount advice more than non‐experts,^23^ despite decision‐making research showing that using advice increases accuracy.^13^, ^24^ One of the potential drawbacks of experts discounting advice is that, although they may make more accurate domain‐specific estimates, they might also be overly confident in their knowledge in the domain.^25^ One might expect doctors and nurses to be more directly involved with prognostication than for instance allied health professionals. This could potentially lead to more familiarity with, and expertise in, prognostication and thus more discounting of advice. Therefore, it is relevant to also consider the impact of clinical profession and experience when investigating how people take advice into account in prognostic decision‐making tasks.

This paper details a Randomised Controlled Trial (RCT) involving palliative healthcare professionals (HCPs) completing an online decision‐making task involving predicting advanced cancer patients' two‐week survival, where one group received advice perceived to come from another HCP and the other group received advice perceived to come from the PiPS‐B14 prognostic algorithm.^26^ To our knowledge, there has been no previous research specifically addressing the question of how clinicians integrate prognostic advice from colleagues or from algorithms, and what factors potentially influence advice integration.

## 2 AIMS

### 2.1 Primary aim

The primary study aim was to assess the level to which clinicians integrate advice perceived to be from either another clinician or an algorithm into their estimates of the prognosis of palliative care patients.

### 2.2 Secondary aims

The secondary aims explored the extent to which clinicians' integration of advice were influenced by:

1. Characteristics of the participant (i.e., profession and years of experience)
2. Strength of the prognostic advice received

## 3 METHODS

This trial was reported following the CONSORT 2010 guidance.^27^ Approval from the UCL Research Ethics Committee was obtained (17031/001). The study was prospectively registered on ClinicalTrials.gov (NCT04568629), where the study protocol can be accessed.

### 3.1 Study design

This was an online double‐blind RCT using 1:1 allocation ratio. The study adopted the Judge‐Advisor System (JAS) methodology used in studies of advice‐taking and decision‐making.^28^ A key element in JAS is the differentiation between the roles of the judge and advisor; the advisor offers advice while decision‐making power rests with the judge. Palliative HCPs were asked to provide probability estimates for two‐week survival for five advanced cancer patients before and after receiving advice from an advisor.

### 3.2 Participants

Clinicians were eligible for the study if they worked in adult palliative care and were willing and able to provide written informed consent. All clinicians regardless of professional background were eligible to participate in order to capture the variety of staff who might be part of the multidisciplinary team and involved in patient care.

The study was open to recruitment from October 2020 to April 2021. Participants were recruited through online educational seminars and via email contact through non‐NHS hospices in the UK.

### 3.3 Sample size

Based on calculations from a similar study^19^ and relevant guidelines,^30^ a minimum sample size of 100–200 participants was deemed sufficient to detect a significant difference (with 95% confidence) between the mean WOAs of the two study arms, assuming that the standard deviation of the WOA were to be between 0.3 and 0.4^19^ and the mean difference were to be between 0.085 and 0.155. Further information about this calculation can be found in the study protocol (ClinicalTrials.gov NCT04568629). Due to uncertainty in the assumptions that contributed to the sample size estimates, recruitment continued for six months despite the minimum target being exceeded.

### 3.4 Randomisation

The study website automatically assigned participants to study arms according to a blocked randomisation list (with blocks of size 4, 6 and 8). Study vignettes were presented in random order.

### 3.5 Blinding

The research team and participants were blind to intervention allocation, while the database specialist (CT) was not blinded to allocation. Group allocation was only revealed once the database had been locked and analyses had been completed.

### 3.6 Procedure

Participants accessed an online platform, created by a website developer (CT) for the purpose of the study. After obtaining consent, they were asked to provide relevant demographic and clinical information about themselves (profession, age, gender, work environment, country of employment, years of overall experience and years of palliative care experience).

They then reviewed five patient summaries (‘vignettes’), containing relevant prognostic information (see Appendix S1 for the study vignettes). The vignettes were constructed from anonymised patient data obtained from the PiPS2 study.^26^ The PiPS2 study database contains a variety of prognostic information prospectively collected from patients with advanced cancer and their subsequent length of survival. In order for the five vignettes to represent patients with differing levels of prognostic uncertainty we selected patients with differing estimated survival probabilities (50%; 75% and 90%) using the PiPS‐B14 algorithm. In addition, the vignettes were chosen to represent both men and women with a range of diagnoses, ages and other distinctive features.

After reviewing each vignette:

1. Participants were asked to estimate the probability that the patient would survive the next 2 weeks (estimate on a 0%–100% scale; 0% ‘certain to die’ to 100% ‘certain survival’).
2. Participants were then provided with advice (a probability estimate [i.e., 50%, 75% or 90%]) with the perceived source of advice varying depending on study arm.
3. Participants were asked to provide a final probability estimate.

The study website, procedures and vignettes were piloted on September 2020 by four clinicians working in the Marie Curie Palliative Care Research Department, UCL.

### 3.7 Intervention

Participants were randomly assigned to one of two study arms. The algorithm arm was informed that prognostic advice came from the PiPS‐B14 prognostic tool. PiPS‐B14 is a validated prognostic algorithm that has been shown to be as accurate as an agreed multi‐professional survival estimate.^29^ The study team assumed that the PiPS‐B14 may be unfamiliar to participants. For this reason, participants were further informed that in a previous study the PiPS‐B14 risk categories for predicting two‐week survival were as accurate as a doctor's or a nurse's prediction. The clinician arm was informed that advice came from another clinician. Doctors were told that advice was from a nurse, whereas nurses and other types of HCPs were told that advice was from a doctor. The advice was identical in both arms (from PiPS‐B14), and was based on anonymous data collected as part of a previous study.^26^ Depending on the vignette, participants received advice that there was an estimated 50%, 75%, or 90% probability of death within 14 days (2/5 vignettes were 90% estimates; 2/5 were 75%; 1/5 was 50%). Participants in the clinician arm were not aware that advice actually emanated from PiPS‐B14. This low‐level deception was necessary to determine the impact of the source of advice on participants' prognostic estimates.

### 3.8 Outcome measure

The primary outcome was participants' initial and final probability estimates of the two‐week survival.

### 3.9 Analysis

The statistical analysis plan is available on ClinicalTrials.gov (NCT04568629). A per‐protocol analysis was conducted, including only those who completed the study. Demographic details about participants were described using descriptive statistics. All analyses were repeated and completed while blinded, using Stata version 15 or above.

For each vignette, Weight of Advice (WOA) was calculated. This is a measure of the extent to which participants change judgement in light of advice received, and is a standard analytical approach in psychological experiments of this type. WOA is calculated by comparing participants' final estimate (f) against their initial estimate (i) and the advice (a) provided; WOA $=\left|f-i\right|/|a-i|$.^23^ WOA ranges from 0 (100% discounting of advice) to 1 (0% advice discounting).^31^

Following previous research using a similar methodology,^32^ WOA values higher than 1 were capped at 1. In cases where participants' initial estimates were the same as the advice received, it was not possible to compute a WOA score, and such instances were excluded from analysis.

Data were summarised using descriptive statistics (means and standard deviations [SDs]). Multilevel linear regression analyses were conducted to compare mean WOA scores between study arms (primary aim) accounting for repeated measures within each participant and further regression analyses included clinician descriptors and strength of advice (secondary aims). A non‐parametric (sensitivity) analysis was also conducted. In line with previous research,^33^ five WOA categories were created (<0.2; 0.2–<0.4; 0.4–<0.6; 0.6–<0.8; 0.8–1), and a multilevel ordinal logistic model was performed.

## 4 RESULTS

**FIGURE 1:**
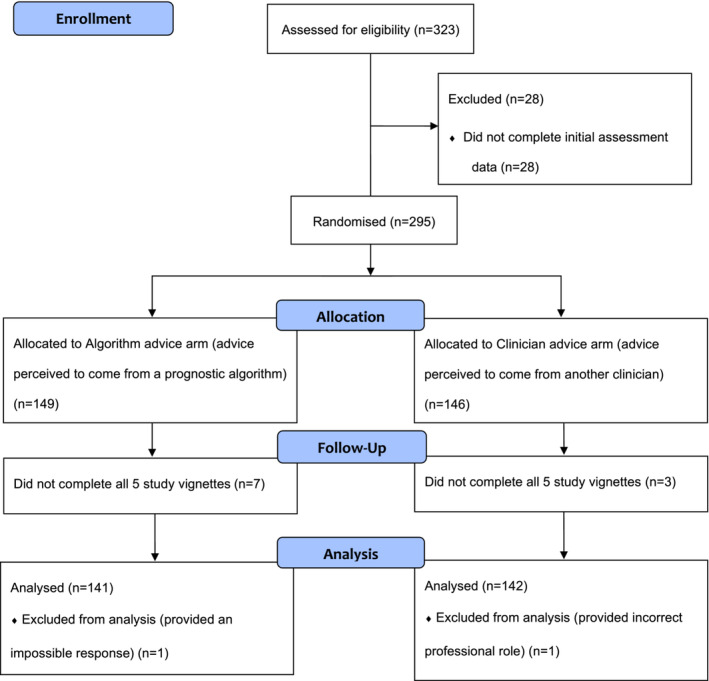
Consort flow diagram

Two hundred and eighty‐three of 323 (87%) enrolled participants completed the study and were included in the analysis. Forty participants were excluded: Thirty eight participants did not complete all five vignettes, and two participants were excluded for other reasons (see Figure 1). Participants' demographics are summarised in Table 1.

**TABLE 1:** Participants' demographic information

	Total		
	*n* (%)	Algorithm arm	Clinician arm
Sample size	283 (100)	141 (50)	142 (50)
Profession			
Doctors	138 (49)	70 (49)	68 (48)
Nurses	116 (41)	56 (40)	60 (42)
Other healthcare professionals	29 (10)	15 (11)	14 (10)
Occupational therapist	6 (21)	3 (20)	3 (22)
Physiotherapist	7 (24)	5 (33)	2 (14)
Healthcare assistant	7 (24)	3 (20)	4 (29)
Other or not specified^a^	9 (31)	4 (27)	5 (35)
Age (years)			
21–30	19 (7)	9 (6)	10 (7)
31–40	67 (24)	32 (23)	35 (25)
41–50	109 (38)	52 (37)	57 (40)
51+	88 (31)	48 (34)	40 (28)
Gender^b^			
Male	23 (8)	12 (9)	11 (8)
Female	260 (92)	129 (91)	131 (92)
Work environment			
Hospice	157 (55)	78 (55)	79 (56)
Hospital	27 (10)	14 (10)	13 (9)
Community	28 (10)	12 (9)	16 (11)
Other	2 (1)	1 (1)	1 (1)
Multiple settings	69 (24)	36 (25)	33 (23)
Country of employment			
GBR	266 (94)	132 (94)	134 (94)
Other countries^c^	17 (6)	9 (6)	8 (6)
Years of experience			
≤14	100 (35)	51 (36)	49 (35)
15+	183 (65)	90 (64)	93 (65)
Years of palliative care experience			
≤5	85 (30)	39 (28)	46 (32)
6–14	102 (36)	51 (36)	51 (36)
15+	96 (34)	51 (36)	45 (32)

^a^ Includes the following roles: Social worker; Pharmacist; Speech and Language Therapist; AHP (Allied Health Professional); Deputy Head of Inpatients; Advanced Clinical Practitioner.

^b^ Gender categories included ‘other’ or ‘prefer not to say’, however no participants chose these options and therefore not listed in the table.

^c^ Includes the following countries: Hong Kong; Ireland; Gibraltar, Spain; United Arab Emirates; United States of America.

### 4.1 Primary analysis (integration of advice from clinician or algorithm)

Participants' initial and final probability estimates and WOA are described in Table 2.

**TABLE 2:** Participants' overall probability estimates and WOA scores

	Initial estimate			Final estimate			WOA		
	Overall	Algorithm	Clinician	Overall	Algorithm	Clinician	Overall	Algorithm	Clinician
	Mean (SD)								
Overall	64.93 (19.07)	64.56 (18.87)	65.30 (19.28)	69.19 (16.97)	69.19 (16.52)	69.20 (17.42)	0.37 (0.35)	0.44 (0.36)	0.31 (0.33)
Analysis	WOA difference						−0.12		
	95% CI						−0.18, −0.07		
	*p*‐value						<0.001		

As evident in the table, participants in the clinician advice arm had a significantly lower WOA score than participants in the algorithm advice arm (WOA difference = −0.12 [95% CI ‐0.18, −0.07], *p* < 0.001). The sensitivity analysis confirmed the results, and the difference between study arms remained statistically significant (*p* < 0.001).

### 4.2 Secondary analyses (other factors influencing clinicians' integration of advice)

Table 3 describes the WOA by the characteristics of the participant (profession, total and palliative care experience) and the strength of the prognostic advice received by study arm.

**TABLE 3:** WOA and interaction between clinicians' demographics, strength of advice and study arm

	WOA		
	Algorithm	Clinician	WOA difference
	Mean [95% CI]		
Profession			
Doctor	0.40 [0.35, 0.46]	0.27 [0.22, 0.32]	−0.13 [−0.20, −0.07]
Nurse	0.46 [0.40, 0.51]	0.32 [0.27, 0.38]	−0.13 [−0.22, −0.05]
Other HCP	0.52 [0.40, 0.63]	0.48 [0.37, 0.60]	−0.03 [−0.24, 0.17]
Total experience (years)			
≤14	0.43 [0.37, 0.50]	0.29 [0.23, 0.36]	−0.14 [−0.24, −0.04]
15+	0.44 [0.39, 0.49]	0.32 [0.28, 0.37]	−0.11 [−0.18, −0.05]
Palliative care experience (years)			
≤5	0.49 [0.42, 0.56]	0.37 [0.30, 0.43]	−0.12 [−0.22, −0.02]
6–14	0.40 [0.33, 0.46]	0.30 [0.24, 0.36]	−0.10 [−0.19, −0.00]
15+	0.44 [0.38, 0.50]	0.27 [0.21, 0.34]	−0.16 [−0.25, −0.08]
Strength of advice received (probability estimate)			
50%	0.48 [0.42, 0.54]	0.31 [0.25, 0.37]	−0.16 [−0.24, −0.08]
75%	0.36 [0.32, 0.41]	0.27 [0.22, 0.31]	−0.09 [−0.16, −0.03]
90%	0.50 [0.45, 0.55]	0.37 [0.32, 0.42]	−0.13 [−0.20, −0.06]

‘Other’ types of HCPs had higher WOA scores (0.50 [95% CI 0.42, 0.58]) than nurses (0.39 [95% CI 0.35, 0.43], WOA difference = −0.11 [95% CI ‐0.20, −0.21]) and doctors (0.34 [95% CI 0.30, 0.37], WOA difference = −0.16 [95% CI ‐0.25, −0.07]). There was no interaction between profession and study arm on WOA (*p* = 0.150) (see Figure 1 in Appendix S2).

Participants with total experience of 15+ years had a slightly higher WOA score (0.38 [95% CI 0.35, 0.41]) than less experienced staff (0.36 [95% CI 0.32, 0.41], WOA difference = 0.02 [95% CI ‐0.04, 0.07]). There was no interaction between experience and study arm on WOA (*p* = 0.935) (see Figure 2 in Appendix S2).

WOA scores of participants with up to 5 years of palliative care experience were higher (0.43 [95% CI 0.38, 0.48]) than those with 6–14 years of palliative care experience (0.35 [95% CI 0.30, 0.39], WOA difference = −0.08 [95% CI ‐0.15, −0.015]) and those with over 15 years of experience (0.36 [95% CI 0.31, 0.40], WOA difference = −0.07 [95% CI ‐0.14, −0.01]). There was no interaction between palliative care experience and study arm on WOA (*p* = 0.152) (see Figure 3 in Appendix S2).

Advice given as a 75% probability estimate had a lower WOA score (0.31 [95% CI 0.28, 0.35]) than advice received at either the 50% (0.40 [95% CI 0.35, 0.44], WOA difference = 0.08 [95% CI 0.04, 0.12]) or 90% level (0.43 [95% CI 0.40, 0.47], WOA difference = 0.12 [95% CI 0.09, 0.15]). The overall interaction between strength of advice and study arm on WOA was significant (*p* < 0.001) (see Figure 4 in Appendix S2).

## 5 DISCUSSION

### 5.1 Main findings

In an online RCT, we have found that palliative care professionals (regardless of their professional background, healthcare experience or years spent specifically working in palliative care) integrated prognostic advice more when they perceived it to be coming from an algorithm rather than from another clinician, when prognosticating imminent death.

### 5.2 Discussion in relation to other findings

Our finding that clinicians integrate advice from a prognostic algorithm more than advice from other clinicians is in line with evidence suggesting that prognostic algorithms can be helpful in clinical practice. Evidence has shown that algorithms are sometimes used as confirmatory tools, validating clinicians' predictions, correcting prognostic impressions, or overcoming tendencies to ignore or overestimate prognoses.^21^ Use of prognostic algorithms may increase clinicians' confidence and thereby encourage communication of prognostic information and its use in clinical decision‐making.^21^ Evidence suggests that prognostic algorithms could be used as educational tools, especially for less experienced staff.^22^ Moreover, such models can increase prognostic authority by reducing ambiguity in cases of disagreement.^21^, ^22^ A scoping review found that doctors are sometimes perceived as the final decision‐maker overruling other staff's opinions or assessments when making prognostic decisions^34^, and prognostic algorithms could serve as an objective external opinion in cases of professional disagreement.

It is interesting to note that clinicians integrated advice perceived to be coming from an algorithm more than advice perceived to be coming from a clinical colleague, even though they were informed that such advice was as accurate as a doctor's or a nurse's prediction. As noted previously, prognostic algorithms have not consistently shown superiority to clinical prediction of survival;^16^ therefore, clinicians should exercise due caution when evaluating how much confidence to place in their predictions.

In our study, we found that participant experience did not impact how clinicians integrated advice. Despite the fact that doctors and nurses might be more involved and experienced in making survival predictions, there is conflicting evidence regarding whether experience improves prognostic accuracy, and there is no clear evidence about whether some types of HCP are better prognosticators than others.^6^ In this study, there was no significant difference between participants' level of experience and their WOA.

The only factor that had a significant impact on participants' advice integration was the strength of the advice. When participants received the advice that a patient had a 75% probability of surviving 2 weeks, they were less likely to integrate the advice into their estimates compared to advice given with lower or higher strength. The reasons for this finding are unclear and may have been a statistical anomaly. Future research should aim at exploring this and provide plausible explanations, for example if there are potential biases towards certain (even) estimates or whether participants' averaging strategies could explain this.

Our results do not shed light on the optimal strategy for combining initial prognostic judgements with advice. Our study did not allow for study participants to share their thoughts or reasoning behind their decisions. Future studies should aim at exploring this in greater detail. There is a need for more research on understanding human‐algorithm interaction^17^ and clinicians' prognostic decision‐making processes.^15^ If prognostic algorithms are to find a place in palliative care clinical practice, then it is important to understand how they are used by clinicians and to ensure that their outputs are used appropriately, neither treated with undue scepticism nor with misplaced trust. Moreover, this study explored how professionals integrate advice from a colleague with a different professional background than themselves. Future studies could explore whether other attributes of the advisor (e.g., profession, age, gender, ethnicity or years of experience) may influence advice integration.

### 5.3 Strengths and limitations

This study involved 283 palliative care professionals completing a prognostic decision‐making task. The study followed a rigorous RCT study design, and analyses were conducted blinded, adding robustness to the findings. The experimental design allowed us to control for several variables involved in prognostic decision‐making, which meant we were able to isolate and quantify the degree to which clinicians' integration of advice were influenced by the source of the advice. It would have been difficult to achieve the same quantitative outcome, had the study relied on observations or interviews involving participants' strategies or attitudes towards integrating prognostic advice from colleagues or algorithms.

There were some limitations with the experimental design. By necessity, the experimental set‐up was rather artificial and therefore lacked some ecological validity. Study vignettes were based on information from palliative care patients, collected as part of the PiPS2 study. This meant that some clinical information such as rate of decline was not available, which may have been useful in prognostic decision‐making. Also, most clinicians would usually expect to review a patient face‐to‐face before providing a prognostic estimate. Nonetheless, there are occasions when prognostic decisions might be made without face‐to‐face review (e.g. deciding on admission priorities to a hospice on the basis of information provided in referral forms).

This study focused on patients' two‐week survival rather than survival to other time points. This was because the PiPS‐B algorithms and database which were used to create the vignettes are only able to calculate survival probabilities at either 14 or 56 days. Of these two time points, we were most interested in 14 days survival as we considered this to be the more clinically useful time point (identifying for instance those patients who may or may not be suitable for hospice admission or fast‐track arrangements for discharge home for terminal care). Future studies could explore the influence of other time frames, and whether these have an impact on clinicians' integration of advice.

Another limitation of the study set‐up was the formulation of patients' survival in percentage probability estimates. Clinicians might not formulate prognoses using precise probability estimates, instead other formulations might be used to manage and mitigate prognostic uncertainty.^35^, ^36^ Therefore, the clinical (rather than statistical) importance of the differences that we observed between the clinicians' integration of algorithmic or human advice is unclear. Future research should aim at studying how prognostic advice is actually used in clinical practice.

Study participants were primarily doctors and nurses, while ‘other’ types of HCPs only represented 10% of the overall sample. This raises issues related to comparing the other types of HCPs to other professions. Since the hospices circulated the invitation email to their clinicians, we do not know how many clinicians were approached in total and if any selection bias was present. Future studies should aim at recruiting more other types of HCPs, creating a more balanced cohort of professional backgrounds.

The magnitude of difference between the WOA in those receiving advice perceived to come from an algorithm as opposed to from another clinician was −0.12 (95% ‐0.18, −0.07). Although statistically significant, the clinical importance of this difference is not immediately apparent. There is no agreed minimally important clinical difference in WOA, and further research would be needed to fully understand the effect (if any) that these differences would have on real‐world clinical judgements or management plans.

## 6 CONCLUSION

Our data show that, in an online RCT using patient vignettes, clinicians integrate advice more, if it is perceived to come from a prognostic algorithm rather than from another clinician. Further studies are needed to understand how prognostic decision‐making is carried out and how prognostic algorithms are used in clinical practice.

## AUTHOR CONTRIBUTIONS

**Andrea Bruun:** Formal analysis (lead); methodology (equal); project administration (lead); writing – original draft (lead); writing – review and editing (lead). **Nicola White:** Formal analysis (supporting); methodology (equal); writing – original draft (supporting); writing – review and editing (supporting). **Linda Oostendorp:** Methodology (equal); supervision (equal); writing – review and editing (supporting). **Victoria Vickerstaff:** Formal analysis (supporting); methodology (equal); writing – review and editing (supporting). **Adam J. L. Harris:** Conceptualization (equal); formal analysis (supporting); methodology (equal); writing – review and editing (supporting). **Christopher Tomlinson:** Data curation (lead); software (lead); writing – review and editing (supporting). **Steven Bloch:** Methodology (equal); supervision (equal); writing – review and editing (supporting). **Patrick Stone:** Conceptualization (equal); funding acquisition (lead); methodology (equal); project administration (supporting); supervision (equal); writing – review and editing (supporting).

## FUNDING INFORMATION

The study was part of a PhD studentship funded by the Marie Curie Chair's Grant MCCC‐FCH‐18‐U.

## CONFLICTS OF INTEREST

Prof Patrick Stone and Dr Victoria Vickerstaff were involved in conducting the PiPS2 study.

## ETHICAL APPROVAL

This study was approved by the UCL Research Ethics Committee (17031/001).

## Supporting information


Appendix S1.

Appendix S2.

## Data Availability

Study data may be shared with other research groups if a reasonable request is submitted to and agreed by the Chief Investigator, Prof Patrick Stone.

## References

[1] Chu C , Anderson R , White N , et al. Prognosticating for adult patients with advanced incurable cancer: a needed oncologist skill. Curr Treat Options Oncol. 2020;21:5. 31950387 10.1007/s11864-019-0698-2 PMC6965075

[2] Chu C , White N , Stone P . Prognostication in palliative care. Clin Med. 2019;19:306‐310. doi:10.7861/clinmedicine.19-4-306 PMC6752241 31308109

[3] Perez‐Cruz PE , Dos Santos R , Silva TB , et al. Longitudinal temporal and probabilistic prediction of survival in a cohort of patients with advanced cancer. J Pain Symptom Manage. 2014;48:875‐882. doi:10.1016/j.jpainsymman.2014.02.007 24746583 PMC4199934

[4] Glare P , Virik K , Jones M , et al. A systematic review of physicians' survival predictions in terminally ill cancer patients. BMJ. 2003;327:195‐198. doi:10.1136/bmj.327.7408.195 12881260 PMC166124

[5] Gwilliam B , Keeley V , Todd C , et al. Prognosticating in patients with advanced cancer‐observational study comparing the accuracy of clinicians' and patients' estimates of survival. Ann Oncol. 2013;24:482‐488. doi:10.1093/annonc/mds341 23028038

[6] White N , Reid F , Harris A , et al. A systematic review of predictions of survival in palliative care: how accurate are clinicians and who are the experts? PLoS One. 2016;11:e0161407. doi:10.1371/journal.pone.0161407 27560380 PMC4999179

[7] Christakis NA , Lamont EB . Extent and determinants of error in doctors' prognoses in terminally ill patients: prospective cohort study. BMJ. 2000;320:469‐472. 10678857 10.1136/bmj.320.7233.469 PMC27288

[8] Maltoni M , Caraceni A , Brunelli C , et al. Prognostic factors in advanced cancer patients: evidence‐based clinical recommendations‐a study by the steering Committee of the European Association for palliative care. J Clin Oncol. 2005;23:6240‐6248. doi:10.1200/jco.2005.06.866 16135490

[9] Kee F , Owen T , Leathem R . Offering a prognosis in lung cancer: when is a team of experts an expert team? J Epidemiol Community Health. 2007;61:308‐313. doi:10.1136/jech.2005.044917 17372290 PMC2652939

[10] Gwilliam B , Keeley V , Todd C , et al. Development of prognosis in palliative care study (PiPS) predictor models to improve prognostication in advanced cancer: prospective cohort study. BMJ. 2011;343:d4920. doi:10.1136/bmj.d4920 21868477 PMC3162041

[11] Harvey N , Fischer I . Taking advice: accepting help, improving judgment, and sharing responsibility. Organ Behav Hum Decis Process. 1997;70:117‐133. doi:10.1006/obhd.1997.2697

[12] Kennedy J , Kleinmuntz DN , Peecher ME . Determinants of the justifiability of performance in ill‐structured audit tasks. J Account Res. 1997;35:105‐123. doi:10.2307/2491456

[13] Yaniv I , Milyavsky M . Using advice from multiple sources to revise and improve judgments. Organ Behav Hum Decis Process. 2007;103:104‐120. doi:10.1016/j.obhdp.2006.05.006

[14] Simmons CPL , McMillan DC , McWilliams K , et al. Prognostic tools in patients with advanced cancer: a systematic review. J Pain Symptom Manage. 2017;53:962‐970.e910. doi:10.1016/j.jpainsymman.2016.12.330 28062344

[15] Hui D , Paiva CE , Del Fabbro EG , et al. Prognostication in advanced cancer: update and directions for future research. Support Care Cancer. 2019;27:1973‐1984. doi:10.1007/s00520-019-04727-y 30863893 PMC6500464

[16] Stone P , Vickerstaff V , Kalpakidou A , et al. Prognostic tools or clinical predictions: which are better in palliative care? PLoS One. 2021;16:e0249763. doi:10.1371/journal.pone.0249763 33909658 PMC8081205

[17] Kelly CJ , Karthikesalingam A , Suleyman M , Corrado G , King D . Key challenges for delivering clinical impact with artificial intelligence. BMC Med. 2019;17:195. doi:10.1186/s12916-019-1426-2 31665002 PMC6821018

[18] Dietvorst BJ , Simmons JP , Massey C . Algorithm aversion: people erroneously avoid algorithms after seeing them err. J Exp Psychol Gen. 2015;144:114‐126. doi:10.1037/xge0000033 25401381

[19] Logg JM , Minson JA , Moore DA . Algorithm appreciation: people prefer algorithmic to human judgment. Organ Behav Hum Decis Process. 2019;151:90‐103. doi:10.1016/j.obhdp.2018.12.005

[20] Yeomans M , Shah A , Mullainathan S , Kleinberg J . Making sense of recommendations. J Behav Decis Mak. 2019;32:403‐414. doi:10.1002/bdm.2118

[21] Hallen SAM , Hootsmans NAM , Blaisdell L , Gutheil CM , Han PKJ . Physicians' perceptions of the value of prognostic models: the benefits and risks of prognostic confidence. Health Expect. 2015;18:2266‐2277. doi:10.1111/hex.12196 24816136 PMC5810722

[22] Stone PC , Kalpakidou A , Todd C , et al. The prognosis in palliative care study II (PiPS2): a prospective observational validation study of a prognostic tool with an embedded qualitative evaluation. PLoS One. 2021;16:e0249297. doi:10.1371/journal.pone.0249297 33909630 PMC8081241

[23] Yaniv I . Receiving other people's advice: influence and benefit. Organ Behav Hum Decis Process. 2004;93:1‐13. doi:10.1016/j.obhdp.2003.08.002

[24] Yaniv I . The benefit of additional opinions. Curr Dir Psychol Sci. 2004;13:75‐78. doi:10.1111/j.0963-7214.2004.00278.x

[25] Larrick RP , Feiler DC . Expertise in decision making. In: Keren G , Wu G , eds. The Wiley Blackwell Handbook of Judgment and Decision Making. Wiley; 2015.

[26] Kalpakidou AK , Todd C , Keeley V , et al. The prognosis in palliative care study II (PiPS2): study protocol for a multi‐Centre, prospective, observational, cohort study. BMC Palliat Care. 2018;17:101. 30103711 10.1186/s12904-018-0352-y PMC6090599

[27] Schulz KF , Altman DG , Moher D . CONSORT 2010 statement: updated guidelines for reporting parallel group randomised trials. BMJ. 2010;340:c332. doi:10.1136/bmj.c332 20332509 PMC2844940

[28] Sniezek JA , Buckley T . Cueing and cognitive conflict in judge‐adviser decision‐making. Organ Behav Hum Decis Process. 1995;62:159‐174. doi:10.1006/obhd.1995.1040

[29] Stone P , Kalpakidou A , Todd C , et al. Prognostic models of survival in patients with advanced incurable cancer: the PiPS2 observational study. Health Technol Assess. 2021;25:1‐118. doi:10.3310/hta25280 PMC8182445 34018486

[30] Machin D , Campbell MJ , Tan SB , et al. Sample Sizes for Clinical, Laboratory and Epidemiology Studies. John Wiley & Sons, Ltd; 2018.

[31] Yaniv I , Kleinberger E . Advice taking in decision making: egocentric discounting and reputation formation. Organ Behav Hum Decis Process. 2000;83:260‐281. doi:10.1006/obhd.2000.2909 11056071

[32] Wang X , Du X . Why does advice discounting occur? The combined roles of confidence and trust. Front Psychol. 2018;9:2381. doi:10.3389/fpsyg.2018.02381 30555394 PMC6282045

[33] Soll JB , Larrick RP . Strategies for revising judgment: how (and how well) people use others' opinions. J Exp Psychol Learn Mem Cogn. 2009;35:780‐805. doi:10.1037/a0015145 19379049

[34] Bruun, A. , Oostendorp, L. , Bloch, S. et al. (2022). Prognostic decision‐making about imminent death within multidisciplinary teams: a scoping review. BMJ Open, 12(4), e057194. doi:10.1136/bmjopen-2021-057194 PMC8984043 35383077

[35] Pino M , Parry R . How and when do patients request life‐expectancy estimates? Evidence from hospice medical consultations and insights for practice. Patient Educ Couns. 2019;102:223‐237. doi:10.1016/j.pec.2018.03.026 29685640

[36] Anderson RJ , Stone PC , Low JTS , et al. Managing uncertainty and references to time in prognostic conversations with family members at the end of life: a conversation analytic study. Palliat Med. 2020;34:896‐905. doi:10.1177/0269216320910934 32233831 PMC7336362

