# Supplementary material for: An online randomised controlled trial of prognosticating imminent death in advanced cancer patients: Clinicians give greater weight to advice from a prognostic algorithm than from another clinician with a different profession
Source: Cancer Med. 2022 Nov 29;12(6):7519–28. doi: 10.1002/cam4.5485 (PMC10067032; doi:10.1002/cam4.5485)
Supplement: Supplementary file 1 — Appendix S1. [file CAM4-12-7519-s001.docx]

**Appendix 1 – Study vignettes**

The study vignettes are included in this appendix. Permission from Victoria Hospice was obtained to use the Palliative Performance Scale v2 (<https://victoriahospice.org/>). At the end of each vignette, the PiPS-B14 probability estimate and actual survival for the patient have been noted. This information was not displayed to participants during the study. The PiPS-B14 probability estimates were used as advice in both study arms (i.e., prognostic algorithm arm and clinician advice arm).

## PRACTICE VIGNETTE

Mr Smith has recently been admitted to the inpatient palliative care unit at St Swithin’s hospice. He has a primary pancreatic cancer with metastases to his lungs and bones. He is currently undergoing palliative radiotherapy.

He is 63 years old and has full capacity. On assessment, there is no evidence of ascites or peripheral oedema. He reports that his eating and drinking are severely reduced, that he has lost weight, but there is no dysphagia. He feels fatigued and is unable to do jobs around the house that he used to do. Sometimes he needs assistance in self-care tasks. He is short of breath. His pulse rate is 88 (beats/min).

His blood tests show the following:

White Blood Count (WBC): 11 x 10^9^/L (normal range 4.0 to 11.0)

Lymphocyte: <1.0 x 10^9^/L (normal range 1.0 to 4.0)

Neutrophil: 10 x 10^9^/L (normal range 1.7 to 8.0)

Platelet: 273 x 10^9^/L (normal range 150 to 450)

Urea: 7 mmol/L (normal range 2.5 to 7.8)

Albumin: 26 g/L (normal range 35 to 50)

Alkaline Phosphatase: 105 U/L (normal range 30 to 130)

Alanine Transaminase: 12 U/L (normal range 0 to 52)

C-Reactive Protein (CRP): 288 mg/L (normal range 0.0 to 10.0)

Lactic acid Dehydrogenases (LDH): 1183 U/L (normal range 140 to 280)

His PPS score is 60% (shown below):


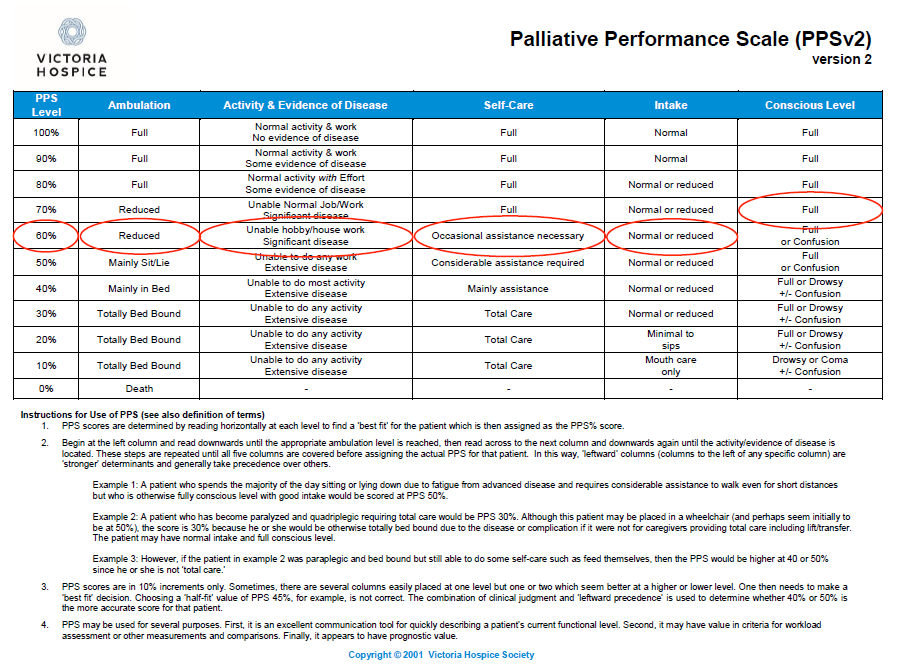


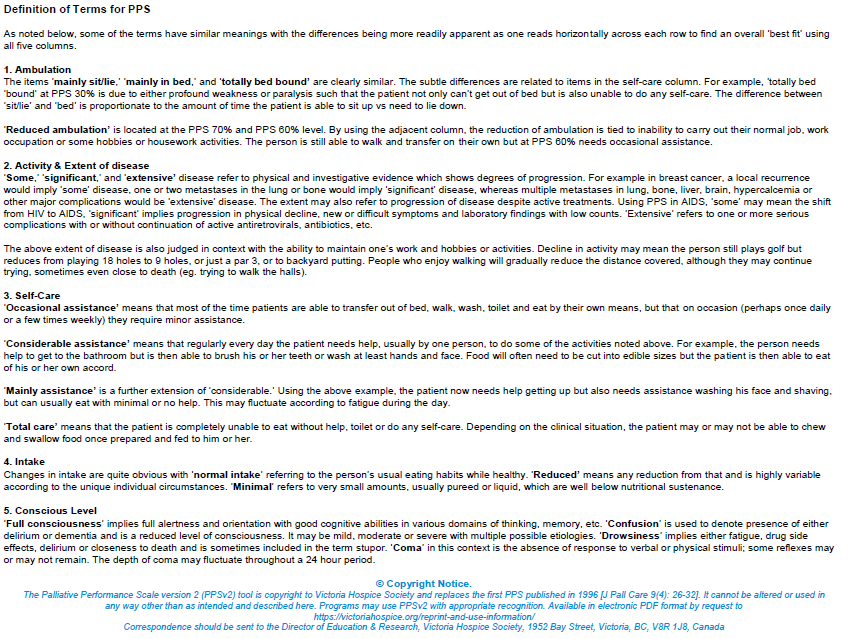


The PiPS-B14 model predicted that the probability of the patient surviving 14 days was 80%.

The patient died 18 days after this assessment.

## VIGNETTE 1

Miss Johnson has recently been admitted to the inpatient palliative care unit at St Swithin’s hospice. She has a primary head and neck cancer with metastases to her bones. She is not currently undergoing any cancer therapy.

She is 54 years old and has full capacity. On assessment, there is no evidence of ascites or peripheral oedema. She reports that her eating and drinking are severely reduced, that she has lost weight, and that she has difficulty swallowing. She feels fatigued and is unable to do activities that she used to do. Sometimes she needs assistance in self-care tasks. She is not short of breath. Her pulse rate is 76 (beats/min).

Selected blood tests show the following:

White Blood Count (WBC): 12 x 10^9^/L (normal range 4.0 to 11.0)

Lymphocyte: <1 x 10^9^/L (normal range 1.0 to 4.0)

Neutrophil: 12 x 10^9^/L (normal range 1.7 to 8.0)

Platelet: 254 x 10^9^/L (normal range 150 to 450)

Urea: 7 mmol/L (normal range 2.5 to 7.8)

Albumin: 37 g/L (normal range 35 to 50)

Alkaline Phosphatase: 108 U/L (normal range 30 to 130)

Alanine Transaminase: 85 U/L (normal range 0 to 52)

C-Reactive Protein (CRP): 107 mg/L (normal range 0.0 to 10.0)

Lactic acid Dehydrogenases (LDH): 317 U/L (normal range 140 to 280)

Her PPS score is 60% (shown below):


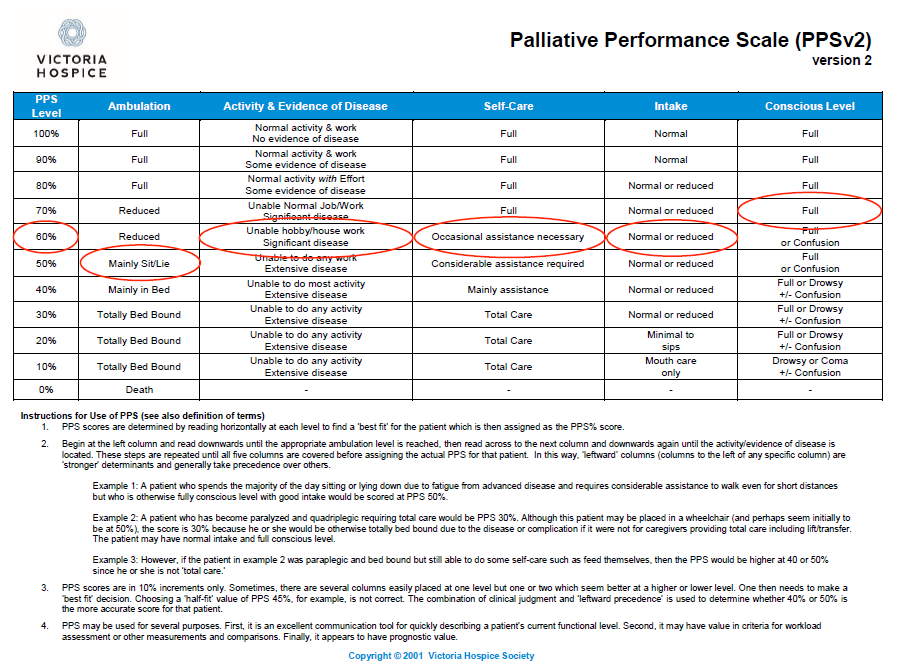


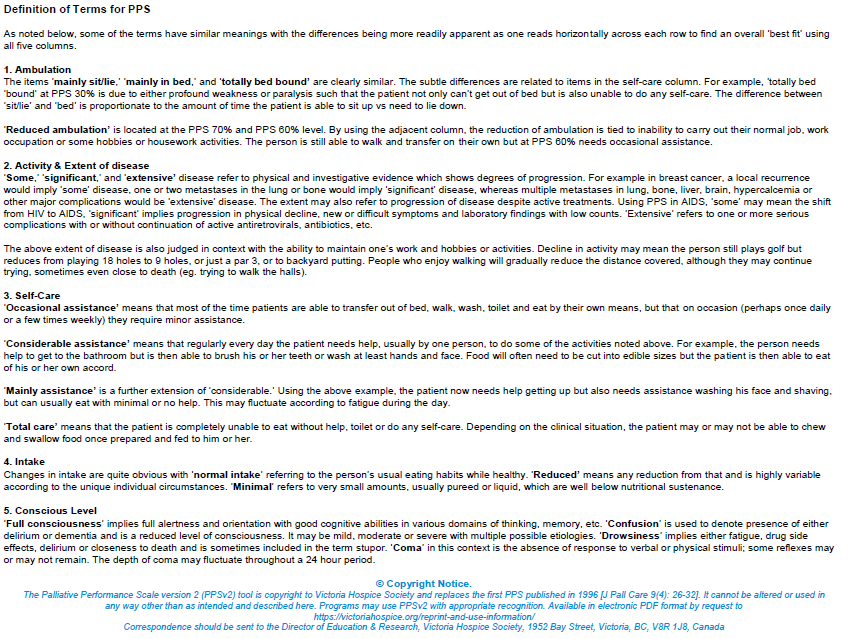


The PiPS-B14 model predicted that the probability of the patient surviving 14 days was 90%.

The patient died 23 days after this assessment.

## VIGNETTE 2

Mrs Williams has recently been admitted to the inpatient palliative care unit at St Swithin’s hospice. She has a gynaecological cancer with metastases to her lungs and bones. She is not currently undergoing any cancer therapy.

She is 60 years old and has full capacity. On assessment, there is no evidence of ascites or peripheral oedema. She reports normal eating and drinking, that she has not lost weight, and that there is no dysphagia. She does not feel fatigued, but she is unable to do most of the activities that she used to do. She needs some assistance in self-care tasks. She is not short of breath. Her pulse rate is 108 (beats/min).

Selected blood tests show the following:

White Blood Count (WBC): 8 x 10^9^/L (normal range 4.0 to 11.0)

Lymphocyte: 1 x 10^9^/L (normal range 1.0 to 4.0)

Neutrophil: 7 x 10^9^/L (normal range 1.7 to 8.0)

Platelet: 103 x 10^9^/L (normal range 150 to 450)

Urea: 5 mmol/L (normal range 2.5 to 7.8)

Albumin: 31 g/L (normal range 35 to 50)

Alkaline Phosphatase: 76 U/L (normal range 30 to 130)

Alanine Transaminase: 14 U/L (normal range 0 to 52)

C-Reactive Protein (CRP): 4 mg/L (normal range 0.0 to 10.0)

Lactic acid Dehydrogenases (LDH): 414 U/L (normal range 140 to 280)

Her PPS score is 40% (shown below):


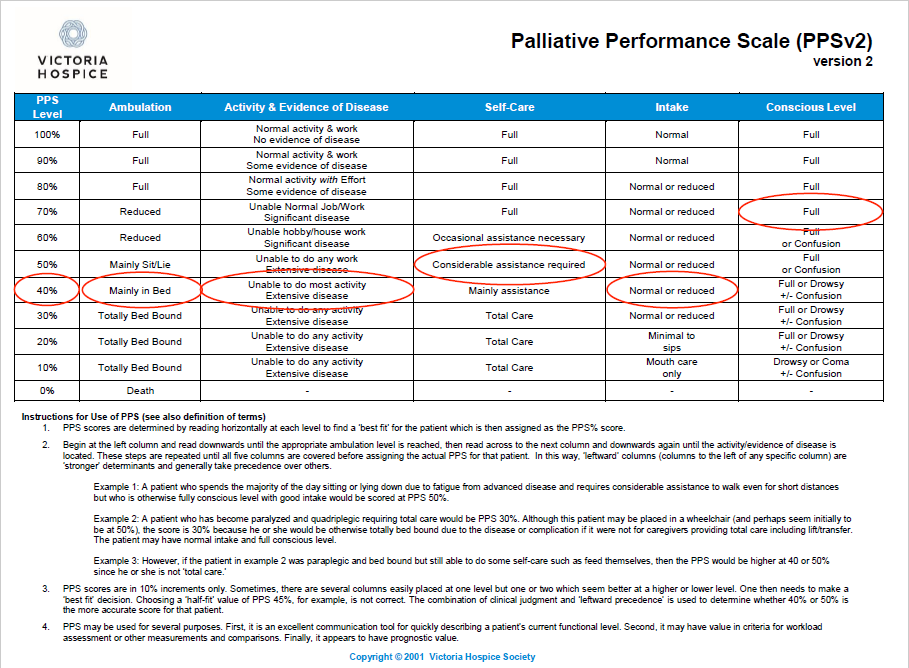


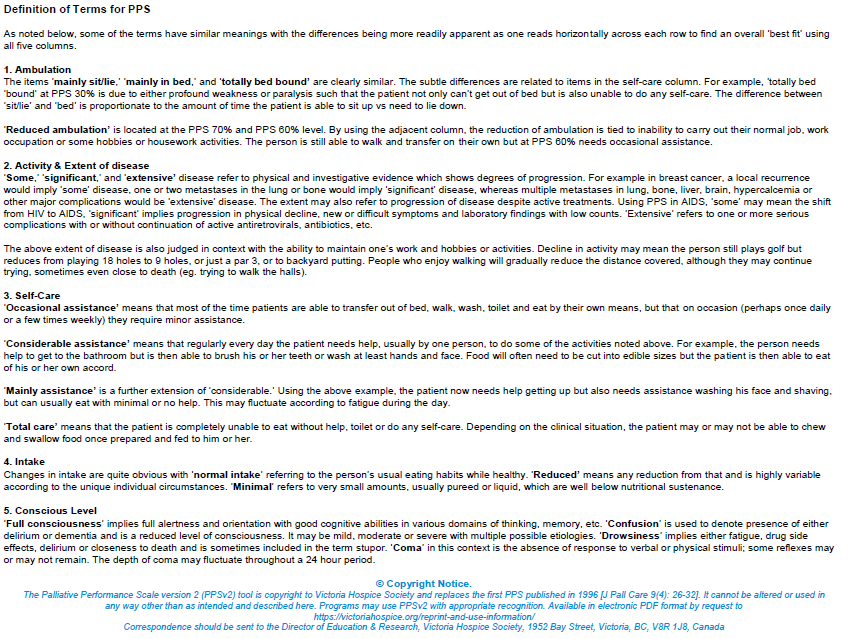


The PiPS-B14 model predicted that the probability of the patient surviving 14 days was 90%.

The patient died 23 days after this assessment.

## VIGNETTE 3

Miss Jones has recently been admitted to the inpatient palliative care unit at St Swithin’s hospice. She has an upper GI primary cancer with nodal metastases and metastases to her liver, lungs and bones. She is not currently receiving tumour-directed therapy.

She is 58 years old and has full capacity. On assessment, there is evidence of ascites and peripheral oedema. She reports that her eating and drinking are severely reduced, that she has lost weight, but there is no dysphagia. She does not feel fatigued, but she is unable to do jobs around the house that she used to do. For the most part, she is still independent in self-care tasks. She is not short of breath. Her pulse rate is 112 (beats/min).

Selected blood tests show the following:

White Blood Count (WBC): 26 x 10^9^/L (normal range 4.0 to 11.0)

Lymphocyte: 2 x 10^9^/L (normal range 1.0 to 4.0)

Neutrophil: 22 x 10^9^/L (normal range 1.7 to 8.0)

Platelet: 90 x 10^9^/L (normal range 150 to 450)

Urea: 6 mmol/L (normal range 2.5 to 7.8)

Albumin: 28 g/L (normal range 35 to 50)

Alkaline Phosphatase: 424 U/L (normal range 30 to 130)

Alanine Transaminase: 29 U/L (normal range 0 to 52)

C-Reactive Protein (CRP): 42 mg/L (normal range 0.0 to 10.0)

Lactic acid Dehydrogenases (LDH): 513 U/L (normal range 140 to 280)

Her PPS score is 60% (shown below):


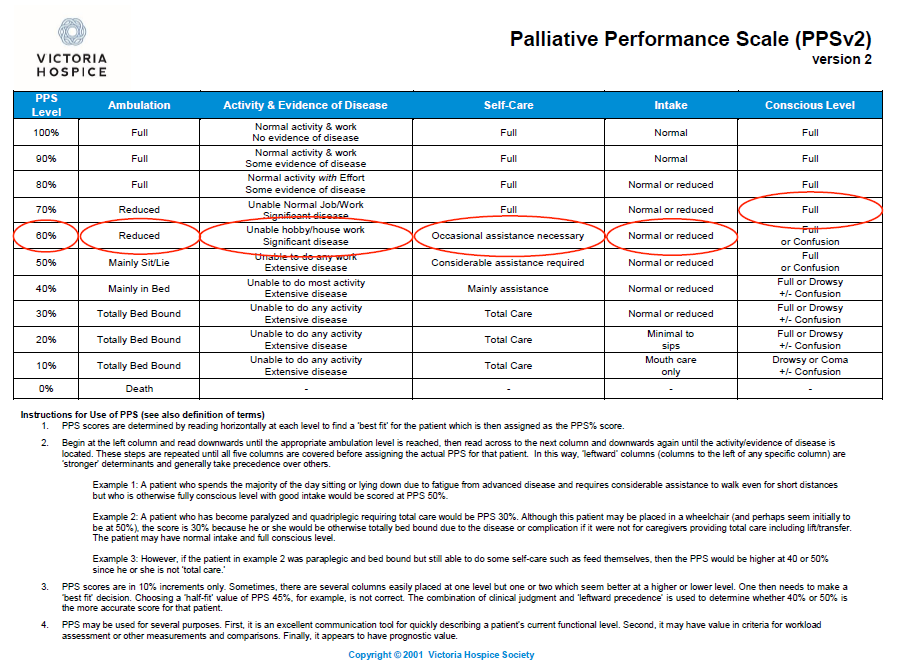


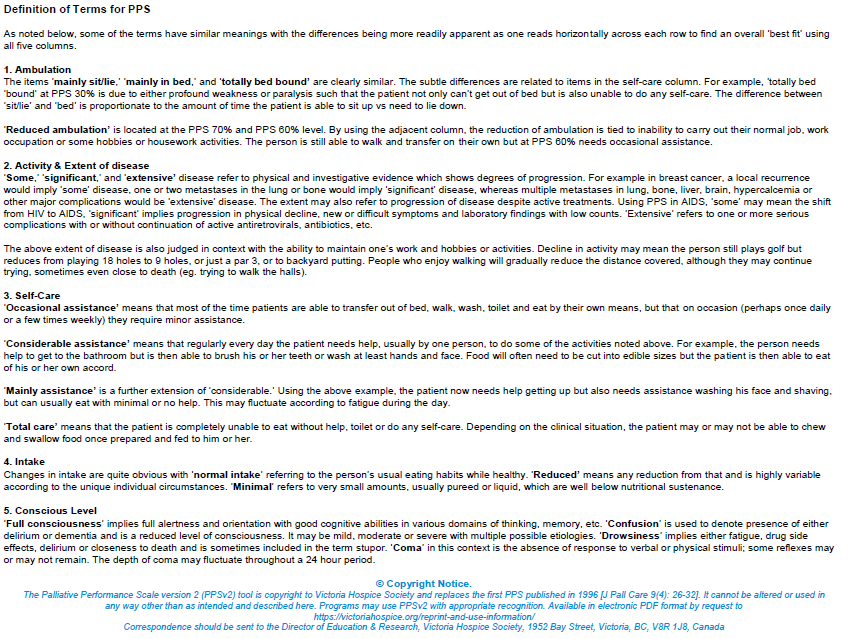


The PiPS-B14 model predicted that the probability of the patient surviving 14 days was 75%.

The patient died 31 days after this assessment.

## VIGNETTE 4

Mr Brown has recently been admitted to the inpatient palliative care unit at St Swithin’s hospice. He has bladder cancer with metastases to lymph nodes and subcutaneous tissues. He is not currently receiving any cancer therapy.

He is 90 years old and has full capacity. On assessment, there is no evidence of ascites but there is evidence of peripheral oedema. He reports that his eating and drinking are moderately reduced, that he has lost weight, and that he has difficulty swallowing. He feels fatigued, and he is unable to do activities that he used to do. Sometimes he needs assistance in self-care tasks. He is short of breath. His pulse rate is 64 (beats/min).

Selected blood tests show the following:

White Blood Count (WBC): 8 x 10^9^/L (normal range 4.0 to 11.0)

Lymphocyte: <1 x 10^9^/L (normal range 1.0 to 4.0)

Neutrophil: 7 x 10^9^/L (normal range 1.7 to 8.0)

Platelet: 321 x 10^9^/L (normal range 150 to 450)

Urea: 37 mmol/L (normal range 2.5 to 7.8)

Albumin: 17 g/L (normal range 35 to 50)

Alkaline Phosphatase: 90 U/L (normal range 30 to 130)

Alanine Transaminase: 9 U/L (normal range 0 to 52)

C-Reactive Protein (CRP): 87 mg/L (normal range 0.0 to 10.0)

Lactic acid Dehydrogenases (LDH): 188 U/L (normal range 140 to 280)

His PPS is 50% (shown below):


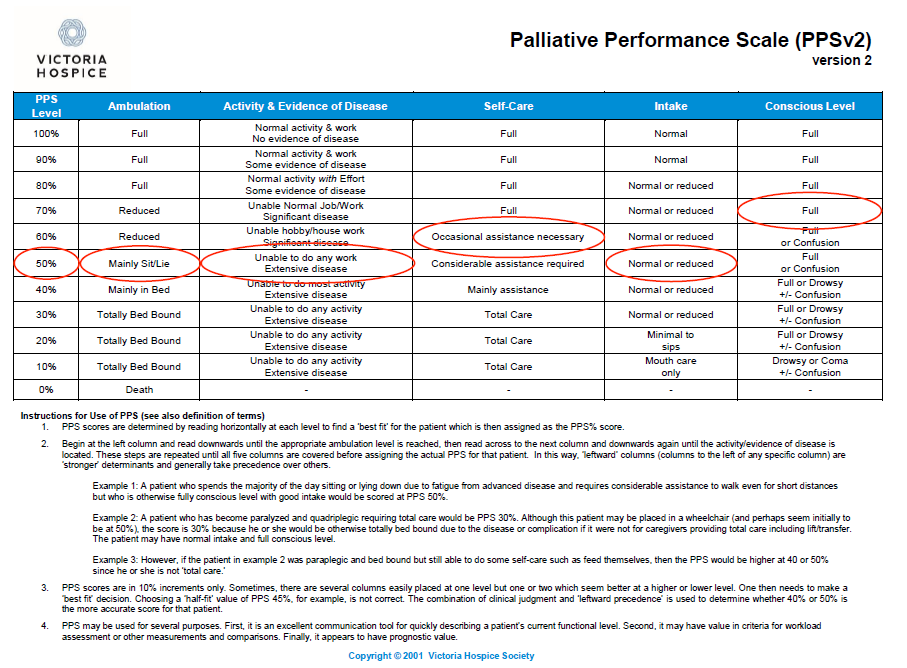


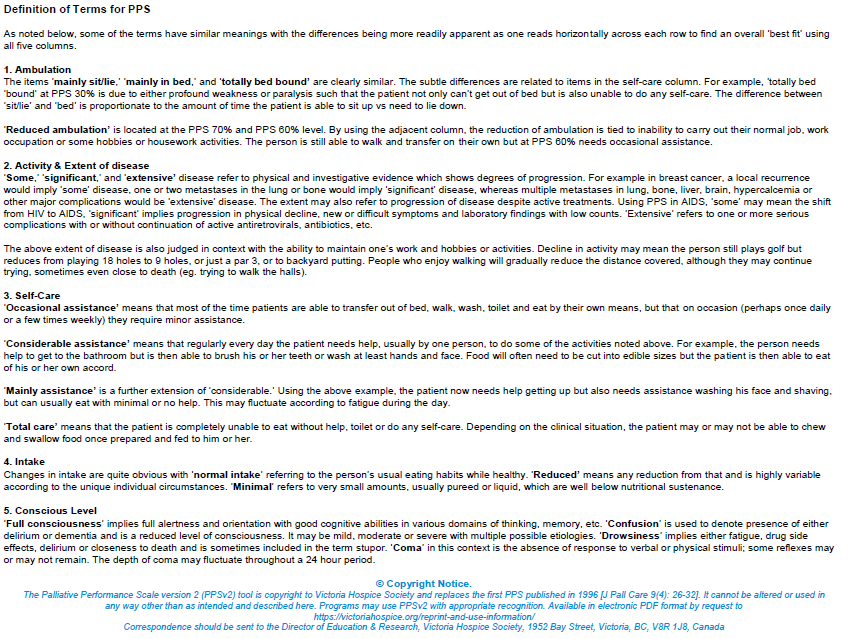


The PiPS-B14 model predicted that the probability of the patient surviving 14 days was 75%.

The patient died 25 days after this assessment.

## VIGNETTE 5

Mr Taylor has recently been admitted to the inpatient palliative care unit at St Swithin’s hospice. He has a lung cancer with nodal spread and metastases to the liver. He is currently not receiving any cancer therapy.

He is 63 years old and has full capacity. On assessment, there is no evidence of ascites but there is evidence of peripheral oedema. He reports that his eating and drinking are severely reduced, that he has lost weight, and that he has difficulty swallowing. He feels fatigued, and he is unable to do activities that he used to do. Sometimes he needs assistance in self-care tasks. He is short of breath. His pulse rate is 95 (beats/min).

Selected blood tests show the following:

White Blood Count (WBC): 7 x 10^9^/L (normal range 4.0 to 11.0)

Lymphocyte: 1 x 10^9^/L (normal range 1.0 to 4.0)

Neutrophil: 6 x 10^9^/L (normal range 1.7 to 8.0)

Platelet: 249 x 10^9^/L (normal range 150 to 450)

Urea: 16 mmol/L (normal range 2.5 to 7.8)

Albumin: 26 g/L (normal range 35 to 50)

Alkaline Phosphatase: 415 U/L (normal range 30 to 130)

Alanine Transaminase: 21 U/L (normal range 0 to 52)

C-Reactive Protein (CRP): 143 mg/L (normal range 0.0 to 10.0)

Lactic acid Dehydrogenases (LDH): 1061 U/L (normal range 140 to 280)

His PPS is 50% (shown below):


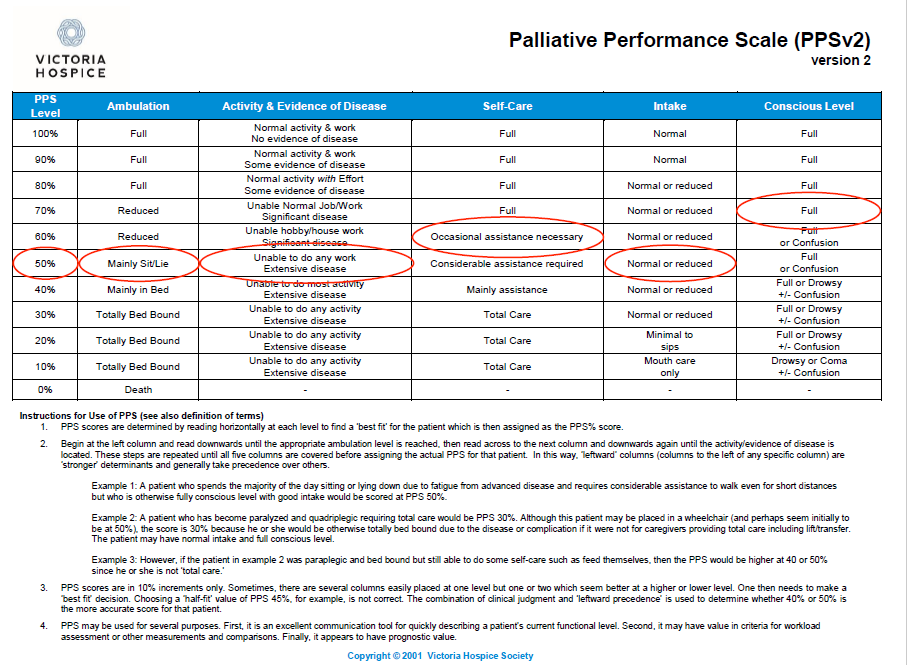


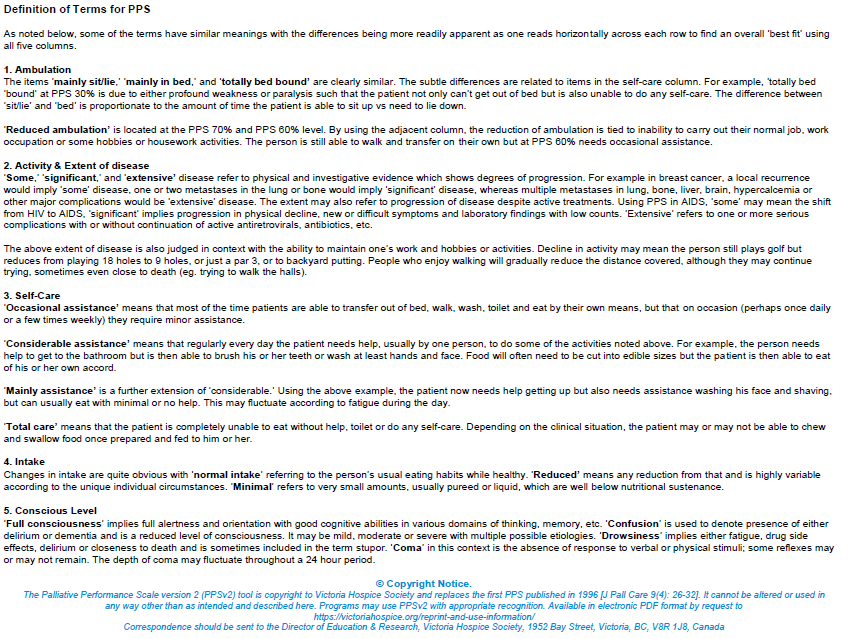


The PiPS-B14 model predicted that the probability of the patient surviving 14 days was 50%.

The patient died 122 days after this assessment.
