# Supplementary material for: An online randomised controlled trial of prognosticating imminent death in advanced cancer patients: Clinicians give greater weight to advice from a prognostic algorithm than from another clinician with a different profession
Source: Cancer Med. 2022 Nov 29;12(6):7519–28. doi: 10.1002/cam4.5485 (PMC10067032; doi:10.1002/cam4.5485)
Supplement: Supplementary file 2 — Appendix S2. [file CAM4-12-7519-s002.docx]

# **Supplementary file 1 - Figures of interactions**

## Figure 1: Interaction between profession and study arm

## Figure 2: Interaction between overall experience and study arm

## Figure 3: Interaction between palliative care experience and study arm

## Figure 4: Interaction between strength of advice and study arm
